# Haplotype-resolved genome assembly of *Coriaria nepalensis* a non-legume nitrogen-fixing shrub

**DOI:** 10.1038/s41597-023-02171-6

**Published:** 2023-05-09

**Authors:** Shi-Wei Zhao, Jing-Fang Guo, Lei Kong, Shuai Nie, Xue-Mei Yan, Tian-Le Shi, Xue-Chan Tian, Hai-Yao Ma, Yu-Tao Bao, Zhi-Chao Li, Zhao-Yang Chen, Ren-Gang Zhang, Yong-Peng Ma, Yousry A. El-Kassaby, Ilga Porth, Wei Zhao, Jian-Feng Mao

**Affiliations:** 1 National Engineering Research Center of Tree Breeding and Ecological Restoration, Beijing Advanced Innovation Center for Tree Breeding by Molecular Design, Key Laboratory of Genetics and Breeding in Forest Trees and Ornamental Plants, Ministry of Education, College of Biological Sciences and Technology, Beijing Forestry University, Beijing, 100083 China; 2 Yunnan Key Laboratory for Integrative Conservation of Plant Species with Extremely Small Populations, Kunming Institute of Botany, Chinese Academy of Sciences, Kunming, 650201 China; 3 Department of Forest and Conservation Sciences, Faculty of Forestry, University of British Columbia, Vancouver, BC V6T 1Z4 Canada; 4 Départment des Sciences du Bois et de la Forêt, Faculté de Foresterie, de Géographie et Géomatique, Université Laval, Québec, QC G1V 0A6 Canada; 5 Department of Ecology and Environmental Science, Umeå Plant Science Centre, Umeå University, Umeå, SE-901 87 Sweden; 6 Department of Plant Physiology, Umeå Plant Science Centre, Umeå University, Umeå, SE-901 87 Sweden

**Keywords:** Plant sciences, Computational biology and bioinformatics

## Abstract

*Coriaria nepalensis* Wall. (Coriariaceae) is a nitrogen-fixing shrub which forms root nodules with the actinomycete *Frankia*. Oils and extracts of *C. nepalensis* have been reported to be bacteriostatic and insecticidal, and *C. nepalensis* bark provides a valuable tannin resource. Here, by combining PacBio HiFi sequencing and Hi-C scaffolding techniques, we generated a haplotype-resolved chromosome-scale genome assembly for *C. nepalensis*. This genome assembly is approximately 620 Mb in size with a contig N50 of 11 Mb, with 99.9% of the total assembled sequences anchored to 40 pseudochromosomes. We predicted 60,862 protein-coding genes of which 99.5% were annotated from databases. We further identified 939 tRNAs, 7,297 rRNAs, and 982 ncRNAs. The chromosome-scale genome of *C. nepalensis* is expected to be a significant resource for understanding the genetic basis of root nodulation with *Frankia*, toxicity, and tannin biosynthesis.

## Background & Summary

*Coriaria nepalensis* Wall. (2n = 40)^1^, also known as Masuri Berry, is a shrub belonging to the genus *Coriaria* of the unigeneric Coriariaceae family, and is mainly distributed in the Himalayan region. *C. nepalensis* is a non-legume nitrogen-fixing plant that forms root nodules with the actinomycete *Frankia*^2,3^. The biological ability of nitrogen-fixation in this species contributes to its rehabilitation capacity of nutrient-poor degraded land^4,5^; in combination with its osmotic adjustment function and drought tolerance^6,7^, *C. nepalensis* improves the abiotic conditions and provides more suitable habitat for associated plant species^8–10^. Furthermore, essential oils and extracts from *C. nepalensis* could be used as promising drugs due to their antimicrobial^11,12^ and anti-convulsant activities^13^. Traditionally, *C. nepalensis* has been used in folk medicine to treat ailments such as toothaches and traumatic injuries^13,14^. The toxic and antibacterial properties of *C. nepalensis* provide an interesting opportunity for the development of a potent new and environmentally friendly pesticide for pest management^15^. Moreover, *C. nepalensis* bark offers an important source of hydrolysable tannin^16,17^, an ideal treatment for tanning leather^16^.

The phylogenetic position of Coriariaceae is still debated^18^. Previous analyses based on plastid *rbcL* gene sequences^19–21^, and the complete chloroplast genome^14^ placed Coriariaceae close to families in Cucurbitales. However, the nuclear genome has not yet been sequenced in Coriariaceae, although the genome assemblies of related taxa, such as in Datiscaceae^22^ and Begoniaceae^23^, have been published.

Molecular genetic investigation of non-legume nitrogen-fixation and root nodulation from *Frankia* requires a high-quality genome assembly and functional annotation of the host plant. Additionally, such genomic resources may also be crucial to advance the phylogenetics of the unigeneric Coriariaceae family and the efficient exploration of *C. nepalensis’* valued natural products.

Here, we report a 620 Mb haplotype-resolved chromosome-scale assembly of *C. nepalensis* using a combination of high-quality PacBio HiFi (High Fidelity) long reads, Illumina reads, and Hi-C sequencing. The genome was assembled with contig N50 length of 11 Mb and 40 haplotype-resolved pseudochromosomes. We predicted 60,862 protein-coding genes, of which 99.5% were functionally annotated. Furtherore, 939 tRNAs, 7,297 rRNAs, and 982 ncRNAs were annotated. The provided genomic resources will be helpful for future functional studies in *C. nepalensis*.

## Methods

### Sample collection, library construction, and genome size estimation

Leave tissue samples for both genome and RNA sequencing were harvested in 2020 from a mature *C. nepalensis* individual growing in Kunming Botanical Garden which was transplanted from Songming county, Kunming, Yunnan province, China. Sampled leaves were immediately flash-frozen in liquid nitrogen and stored at −80 °C until further use. High-quality genomic DNA was extracted from leaf tissue using the DNeasy Plant Mini Kit (QIAGEN, Inc.) and purified using the Mobio PowerClean Pro DNA Clean-Up Kit (MO BIO Laboratories, Inc.). DNA integrity was assessed using Agilent 4200 Bioanalyzer. Messenger RNA (mRNA), whose sequence information was later utilized in protein-coding gene structure prediction, was isolated from leaves using the NEBNext Poly(A) mRNA Magnetic Isolation Module, and RNA quality was determined with the Agilent 2100 BioAnalyzer.

**Fig. 1:**
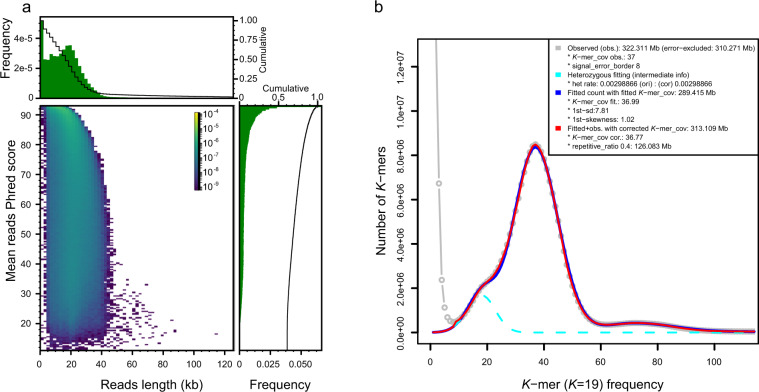
Length and quality of PacBio HiFi reads and genome size survey. (**a**) Reads length and mean Phred score distribution of PacBio HiFi reads. (**b**) 19-mers frequency distribution estimated from PacBio HiFi sequences: observed *K*-mer (raw *K*-mer) frequencies (in grey), fitted *K*-mer frequencies (in blue) with skew normal distribution model, and overall fitting (in red) that concatenated observed and fitted *K*-mer frequencies.

We combined PacBio HiFi long reads sequencing, Illumina sequencing, and Hi-C scaffolding for *C. nepalensis* genome assembly. Genomic DNA fragments were prepared using g-Tubes and purified using AMPure PB beads for library construction and subsequent SMRT cell PacBio HiFi long reads sequencing. Fragment molecules were screened on BluePippin system. The library sequencing was performed on PacBio Sequel II platform, and ccs (https://github.com/PacificBiosciences/ccs) v6.2.0 was used to generate PacBio HiFi data. We obtained ~14.5 Gb (~40×) of HiFi sequencing data with an average length of 19 kb and N50 of 21 kb (Fig. 1a). As for Illumina sequencing, 150 bp paired-end PCR-free libraries were prepared and sequenced on Illumina HiSeq X Ten platform, and ~70 Gb (~200×) of Illumina raw data were obtained. We followed a standard procedure for Hi-C library preparation^24^. In brief, leaf tissues were fixed with formaldehyde and the cross-linked DNA was digested with MboI restriction enzyme. Digested fragments were then biotinylated at 5′ overhangs and joined to form chimeric junctions. After biotin-containing fragments were enriched and sheared, we constructed paired-end sequencing libraries. The Hi-C libraries were sequenced using the Illumina HiSeq X Ten platform and ~67 Gb of Hi-C raw data were obtained. RNA sequencing was performed on Illumina HiSeq X Ten platform after we constructed one sequencing library using the NEBNext Ultra RNA Library Prep Kit, and ~7.5 Gb (50 Mb reads) of raw data were acquired. Then, fastp^25^ software was used for quality control to remove adapters and low-quality and too short Illumina reads (<60 bp). All clean reads were used for further genome assembly and gene predictions.

Genomic characteristics including genome size, repeat content, and heterozygous rate were estimated based on *K*-mer frequencies. Through *K*-mer analysis (*K* = 19) of PacBio HiFi data with Jellyfish^26^ v2.3.0, an overall *C. nepalensis* haplotype genome size of 313.1 Mb was estimated using findGSE v1.94.R^27^ (Fig. 1b).

### *De novo* genome assembly

**Fig. 2:**
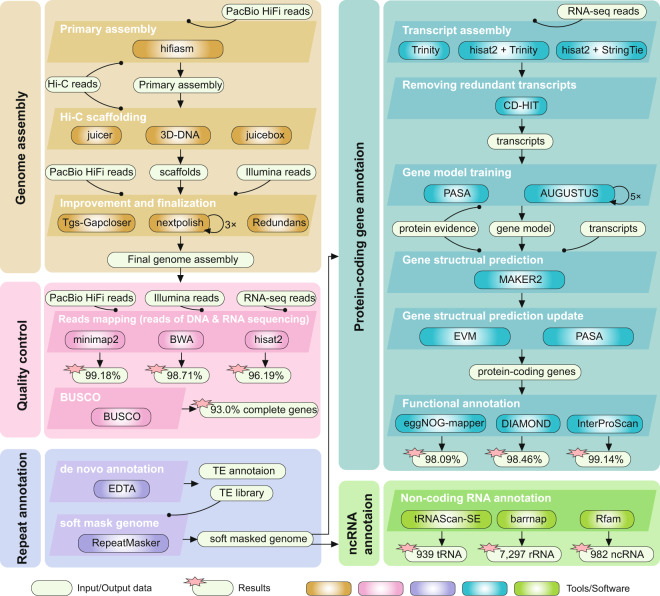
Pipeline overview of genome assembly (yellow), quality control (pink), repeat annotation (purple), protein-coding gene annotation (blue), and ncRNA annotation. Boxes with different color shading represent the different software used in each analytical step.

*De novo* assembly involved three steps: primary assembly, Hi-C scaffolding, and polishing (Fig. 2). With PacBio HiFi reads and Hi-C reads as inputs, we used hifiasm^28^ v0.16.1 to assemble the genome into contigs and obtained a haplotype-resolved assembly with two haplotypes for subsequent analysis. Further, the Hi-C reads that were mapped to the assembly using Juicer^29^ v1.6. 3D-DNA^30^ (-m haploid -i 150000 -r 0--editor-repeat-coverage 5) were then used for preliminary Hi-C assisted chromosome assembly, and Juicebox^31^ (version 201008) was used to manually adjust the chromosome segmentation boundary and any wrong assembly, including switch error. Afterwards, we used 3D-DNA to re-scaffold each chromosome separately and used Juicebox to manually correct any visible error. We used TGS-GapCloser^32^ v1.0.1 (--min_match 1000 –minmap_arg ‘ -x asm20’) to fill the gaps (24 gaps were filled) with HiFi reads and performed three rounds of polishing using NextPolish^33^ v1.4.0 based on Illumina reads, and removed redundant sequences identified by Redundans^34^ v0.13c. Finally, a haplotype-resolved chromosomal level assembly with a total length of 620 Mb was obtained (Table 1). We obtained 40 pseudochromosomes, consistent with the chromosome number reported in a previous karyotype study^1^. We named the chromosomes according to the descendent order of their lengths. Furthermore, as we were describing a haplotype-resolved genome assembly without parental information for subgenome phasing, we arbitrarily denoted the longer one from each pair of homologous chromosomes as haplotype genome “a” (with character “a” in the terminal of the chromosome IDs), while the other chromosome as haplotype genome “b” (with character “b”).

**Table 1:** Statistics of the haplotype-resolved genome assembly of *C. nepalensis*.

Features	Statistics
**Sequencing**	
Raw bases of WGS-PacBio HiFi (Gb)	~14.5
Raw bases of WGS-Illumina (Gb)	~70
Raw bases of Hi-C (Gb)	~67
Raw bases of RNA-seq (Gb)	~7.5
**Assembly**	
Genome size (Mb)	620.52
Number of pseudochromosomes	40
Chloroplast genome assembly (bp)	158,558
Mitochondria genome assembly (bp)	480,951
N50 of contigs (Mb)	10.97
L50 of contig	22
N50 of scaffolds (Mb)	12.9
L50 of scaffolds	11
Number of gaps	62
GC content (%)	34.78
Complete BUSCOs	1,338 (93.0%)
**Annotation**	
Number of protein-coding gene	60,862
Complete BUSCOs	1,440 (97.2%)
Average length of protein-coding gene (bp)	2,892.7
Average length of CDS (bp)	1,324
Average number of exons per transcript	6.3
Number of tRNA	939
Number of rRNA	7,297
Number of unclassified ncRNA	982

**Fig. 3:**
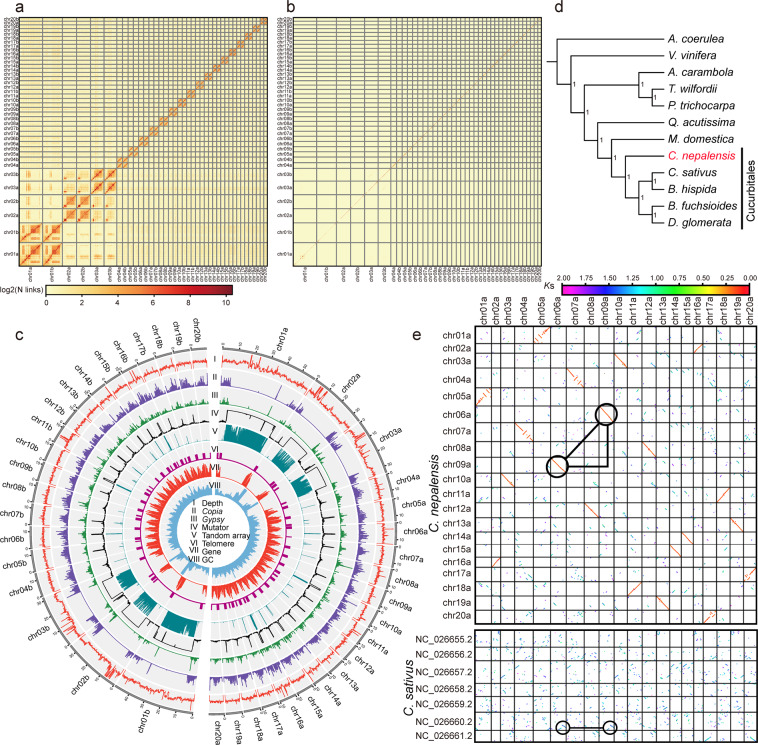
Hi-C density heatmaps, genomic features and evolutionary history of *C. nepalensis*. (**a**) Hi-C chromatin contact density heatmap with a low threshold parameter (minimal mapping quality = 0). (**b**) Hi-C chromatin contact density heatmap with a high threshold parameter (minimal mapping quality = 1). (**c**) Distribution of genomic features of *C. nepalensis*. **I**: sequencing depth distribution of PacBio HiFi reads. **II**–**IV**: The density of *Copia* LTR-RTs, *Gypsy* LTR-TRs and Mutator TE. **V,** **VI**: Distribution of tandem array and telomere sequence. **VII,** **VIII**: Density of protein-coding gene and GC content. (**d**) Phylogenetic tree. (**e**) *K*s dot plots of *C. nepalensis* haplotype genome “a” and *C. sativus*.

Chromosomes chr01-chr03 assemblies were significantly longer than the remaining chromosomes. The assembly of these three pairs of chromosomes was also difficult, showing Hi-C chromatin contact profiles distinct from others (Fig. 3a,b). These three pairs of chromosomes have a large number of gaps (in total 60) in the current assembly, while the other chromosomes had a total of only 2 gaps. Previous karyotype analysis^1^ showed that *C. nepalensis* had three pairs of long chromosomes with extended heterochromatin regions, which is concurrent with the three long chromosomes revealed in the present study. A high number (679,177) of tandem array repeats with the consensus sequence “ATCATTTGCAAGTTATGCACAAAAGTTGTGTCTGTAGTGCAAAACTAGAATTCGTTCGACTTGCTTTGAAATAAGTTATTGACTTGAAATGACTCATTGAAATGATTTTAAGGTTAAACGAATGCACACTTTCCTTGCAATG” was identified on the three long chromosomes chr01-chr03 (Fig. 3c) using TRF^35^ v4.09.1. We found the “TTTAGGG” characterized telomeric sequence in most chromosomes (Fig. 3c), indicating the high quality of our genome assembly.

In addition, a 158,558 bp chloroplast (Pt) genome and a 480,951 bp mitochondrial (Mt) genome were assembled based on short- and long- reads gained from genome sequencing using GetOrganelle^36^ v1.7.5.0 (Table 1).

### Repeat annotation

We performed *de novo* transposable element (TE) annotation using EDTA^37^ v1.9.3 (--sensitive 1 –anno 1) which integrates homology-based and structure-based approaches for TE identification (Fig. 2). A TE library was generated and used for further repeat annotation with RepeatMasker (http://www.repeatmasker.org/RepeatMasker/) (-no_is -xsmall). The output repeat soft-masked genome sequence file was used for gene prediction. A total of 428 Mb (69.0%) of the assembly was annotated as TE (Table 2), of which 61 Mb (9.9%) were long terminal repeat (LTR) retrotransposons. Mutator transposons with 280 Mb (45.2%) in total length showed the highest genome occupation, and also a distribution similar to the high occupation tandem array mentioned above (Fig. 3c). Our further analysis revealed that the sequence motif of these tandem arrays is included inside the Mutator transposons.

**Table 2:** Statistics of repeat annotation of the *C. nepalensis* genome.

Superfamily	Number	Length (bp)	Percent (%)
Class I	102,847	63,608,643	10.25
LTR/Copia	46,213	29,513,519	4.76
LTR/Gypsy	12,994	7,928,273	1.28
LTR/unknown	39,113	24,243,803	3.91
nonLTR/pararetrovirus	730	347,201	0.06
nonLTR/LINE	3,797	1,575,847	0.25
Class II	675,085	314,548,320	50.69
TIR/hAT	17,702	5,852,661	0.94
TIR/CACTA	23,327	7,829,412	1.26
TIR/PIF-Harbinger	17,220	4,264,302	0.69
TIR/Mutator	563,911	280,243,005	45.2
TIR/Tc1_Mariner	9,638	3,344,642	0.54
Helitron	43,287	13,014,298	2.10
Other TEs	432,103	50,184,386	8.09
Total TEs	1,210,035	428,341,349	69.03

### Protein*-*coding genes prediction and other annotations

We collected 139,950 non-redundant protein sequences of the closely related species *Datisca glomerata*^22^, *Begonia fuchsioides*^22^, *Cucumis sativus*^38^, *Vitis vinifera*^39^, *Prunus persica*^40^, and *Arabidopsis thaliana*^41^ as evidence for protein homology (Fig. 2). Three strategies were used to assemble RNA-seq reads into transcripts which were further used as transcriptional evidence for gene annotation. For transcripts assembly, (1) *de novo* assembly was performed using Trinity^42^ v2.13.2; (2) genome-guided assembly was performed using Trinity after reads were mapped to the genome assembly using HISAT2^43^ v2.2.1; and (3) another genome-guided assembly was prepared using StringTie^44^ v2.2.0 with reads mapping using HISAT2. We combined all these three sets of transcripts and obtained 77,555 transcript sequences after removing the redundant sequences with CD-HIT^45^ v4.8.1. Gene structure was annotated using the PASA^46^ v2.5.0 pipeline based on transcriptional evidence. Then, full-length gene sequences were identified by evidence of protein homologies. Based on the full-length gene set, a gene model used for *ab initio* gene structure prediction was trained and optimized using AUGUSTUS^47^ 3.4.0.

Furthermore, the MAKER2^48^ pipeline was used to predict the putative protein-coding gene structure. We performed *ab initio* predictions of gene structures using AUGUSTUS 3.4.0. The transcript evidence and homologous protein evidence were aligned with the genome by BLAST+^49^ v2.11.0 and optimized by exonerate^50^ 2.4.0. AUGUSTUS was used to integrate gene models from the above-mentioned gene prediction. To further improve the annotation accuracy, EVidenceModeler^51^ (EVM) v1.1.1 and PASA were used to integrate and update the gene prediction results. We annotated a final set of 60,862 protein-coding genes (Table 1), among which 30,622 genes were predicted for the haplotype subgenome with a longer set of chromosomes (haplotype genome “a”), and 30,240 genes for the haplotype subgenome “b”. We identified 26,489 putative gene families among *C. nepalensis* (haplotype genome “a”), *Aquilegia coerulea*^52^, *Vitis vinifera*^39^, *Averrhoa carambola*^53^, *Populus trichocarpa*^54^, *Tripterygium wilfordii*^55^, *Malus domestica*^56^, *Datisca glomerata*^22^, *Begonia fuchsioides*^23^, *Benincasa hispida*^57^, *Cucumis sativus*^38^, and *Quercus acutissima*^58^, with OrthoFinder^59^ v2.5.2. (Fig. 3d). Then, 1,199 orthogroups, with a minimum of 83.3% of the species having single-copy genes in any orthogroup, were used to infer the species tree with STAG^60^, and the phylogenetic location of *C. nepalensis* was confirmed. *K*s (synonymous substitutions) dot plots of haplotype genome “a” vs genome “a” and genome “a” vs *C. sativus* were generated with WGDI^61^ v0.62 (Fig. 3e), and one recent unique WGD (whole genome duplication) was revealed and was distinct from that found in *C. sativus*.

BUSCO^62^ was used for evaluating the completeness of the gene set. Out of 1,440 conserved genes, 1,400 (97.2%) were annotated, among which 1,365 (96.9%) were complete and duplicated BUSCO genes.

Three strategies were used for functional annotation of protein-coding genes (Fig. 2, Table 3): (1) we mapped gene sequences against eggNOG^63^ 5.0 database using eggNOG-mapper^64^ v2.1.6 (--target_taxa Viridiplantae) and annotated 98.1% of the genes, of which 55.7 and 49.4% were annotated with GO and KEGG items, respectively; (2) based on the principle of sequence similarity, we annotated 98.5% genes using DIAMOND^65^ v2.0.12 (--evalue 1e-5) against the following four protein databases: Swiss_Prot^66^ (78.2%), TrEMBL^66^ (98.4%), NR^67^ (98.3%), and *Arabidopsis thaliana* genes^41^ (94.1%); (3) we annotated 99.1% of the genes against 14 databases using InterProScan^68^ v5.52–86.0 (Table 3).

**Table 3:** Statistics of protein-coding gene functional annotation.

Method	Database	Number	Percent (%)
eggNOG-mapper	eggNOG	59,758	98.09
	GO	33,948	55.73
	KEGG_KO	30,082	49.38
	KEGG_Pathway	18,595	30.52
	EC	12,864	21.12
	eggNOG	56,164	92.19
	COG	59,758	98.09
DIAMOND		59,981	98.46
	Swiss_Prot	47,641	78.20
	TrEMBL	59,933	98.38
	NR	59,896	98.32
	*A.thaliana*	57,323	94.10
InterProScan		60,396	99.14
	Pfam	51,101	83.88
	CDD	21,628	35.50
	SUPERFAMILY	40,074	65.78
	Interpro	53,945	88.55
	PANTHER	59,108	97.03
	Gene3D	42,846	70.33
	PIRSF	4,336	7.12
	PRINTS	8,886	14.59
	Coils	10,214	16.77
	TIGRFAM	6,982	11.46
	MobiDBLite	26,460	43.43
	TMHMM	14,313	23.49
	Phobius	20,510	33.67
	SMART	19,932	32.72
Total		60,637	99.54

As for non-coding RNA (ncRNA) gene prediction (Fig. 2), we identified 939 tRNAs using tRNAScan-SE^69^ v2.0.8, 7,297 rRNAs using Barrnap v0.9 (https://github.com/tseemann/barrnap) (--kingdom euk), and 982 other ncRNA using Rfam^70,71^ 16.6.

We predicted the genes in the two organelle genomes using OGAP (https://github.com/zhangrengang/OGAP). A total of 131 genes (89 protein-coding genes, 8 rRNAs, and 34 tRNAs) were annotated for the chloroplast genome, and 63 (42 protein-coding genes, 3 rRNAs, and 18 tRNAs) for the mitochondria genome.

### Genome comparison between haplotype assemblies

**Fig. 4:**
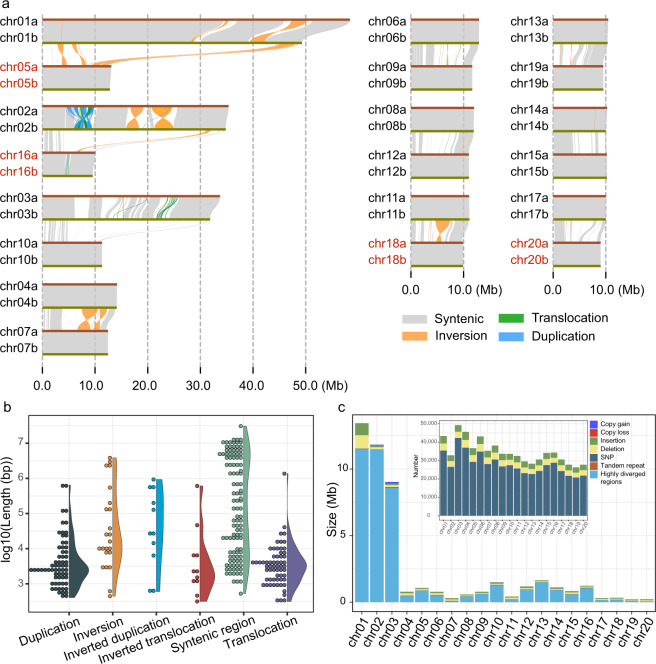
Structural variation and statistics between two haplotype genome assemblies of *C. nepalensis*. (**a**) Structural variation between haplotype genomes. Subgenome “a” (chr01a-chr20a) is used as the reference sequence and subgenome “b” (chr01b-chr20b) is the query. (**b**) Size distributions of different types of structural variation between two haplotype assemblies. (**c**) Numbers and lengths of sequence differences on the syntenic region for each chromosome pair.

The minimap2^72^ v2.24 was used to perform alignments between haplotype assemblies, and SyRI^73^ v1.6 to identify syntenic regions and structural variations (e.g., duplications, inversions, and translocations). Plotsr^74^ v0.5.4 was used for the visualization of the identified structural rearrangements (Fig. 4a). Chr01-chr03 pairs showed remarkable structural variation, while the syntenies of the other homologous chromosome pairs were mostly conserved in high collinearity with only few rearrangements. Syntenic regions were larger than the various types of structural variations (Fig. 4b). Sequence differences (local variation, e.g., SNPs, indels) on syntenic regions were identified (Fig. 4c). Highly diverged regions of long fragments were uneven among chromosome pairs, but the number of sequence differences were minor. Large fragments of collinearity between unpaired chromosomes were also detected (Fig. 4a).

## Data Records

The raw data from PacBio HiFi, Illumina, and Hi-C sequencing were submitted to the SRA database (SRR22412655^75^, SRR22026041^76^, SRR22026042^77^, SRR22026043^78^). The haplotype-resolved genome assembly was deposited at Genbank with accession numbers GCA_027190085.1^79^ and GCA_027186245.1^80^. The genome assembly and gene annotation results of *C. nepalensis* were deposited in the figshare^81^ database.

## Technical Validation

We mapped DNA and RNA sequencing reads to the final genome assembly for evaluation of the assembly quality (Fig. 2). A high read mapping rate of 99.2% was obtained when PacBio HiFi reads were mapped onto the genome using minimap2, and sequencing depth was counted and illustrated in the circos plot in Fig. 3c. We mapped the Illumina reads to the final assembly using BWA^82^ v0.7.17 and obtained a 98.7% reads mapping rate, and a low SNP heterozygosity level of ~0.0027% was obtained after SNPs were identified with SAMtools^83^ v1.13. Furthermore, a single base error rate of ~0.0011% was acquired, and a read mapping rate of 96.2% was obtained when RNA-seq reads were mapped onto the final genome assembly using HISAT2. Since genome coverage by sequencing data was relatively high, our genome assembly has high completeness and continuity.

**Fig. 5:**
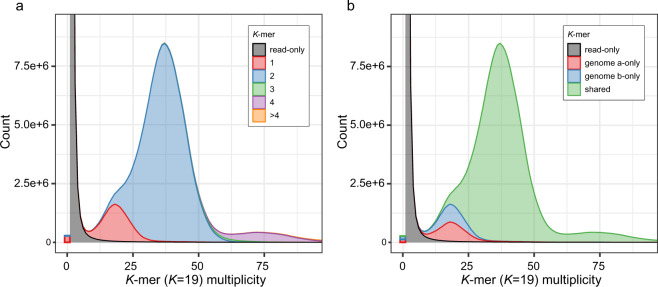
Genome quality assessment with Merqury spectrum plot. (**a)** Copy number spectrum plot for haplotype assemblies of *C. nepalensis*. (**b)** Assembly spectrum plot for evaluating *K*-mer completeness.

We performed further genome assembly quality control with Merqury^84^ analysis (under *K* = 19) (Fig. 5, Table 4) based on PacBio HiFi reads. QVs (consensus quality values) for the individual haplotype genomes “a”, “b”, and shared for both “a” and “b” genomesare 46.39, 45.86, and 46.12, respectively. *K*-mer completeness scores for individual genomes “a”, “b”, and shared for both “a” and “b” genomes are 94.12, 93.68, and 98.87%, respectively. Again, our presented haplotype-resolved genome assembly was confirmed the good quality in completeness.

**Table 4:** Statistics of Merqury analysis for genome quality assessment.

Assembly	QV (quality value)	Error rate	Completeness (%)
Genome “a”	46.39	2.30e-05	94.12
Genome “b”	45.86	2.60e-05	93.68
Genome both “a” and “b”	46.12	2.44e-05	98.87

We further performed BUSCO assessments for the assembly (Table 1), whereit was revealed that complete core genes (including single and multiple copies) accounted for 93.0%, while the missing gene rate accounted for only 4.9%, underscoring the good gene integrity of the assembly.

## Data Availability

All data processing commands and pipelines were carried out in accordance with the instructions and guidelines provided by the relevant bioinformatic software.
